# Proceedings of the Rochester Hahnemannian Society

**Published:** 1889-01

**Authors:** W. H. Baker

**Affiliations:** Secretary


### PROCEEDINGS OF THE ROCHESTER HAHNEMANNIAN SOCIETY.

The regular monthly meeting of the Rochester Hahnemannian Society was held at the office of R. A. Adams, M. D., October 16th, President R. C. Grant, M. D. in the chair.

Members present: Drs. Grant, Biegler, Schmitt, Adams, Carr, Baker; Dr. W. G. Brownell, of this city, and Dr. Walter Johnson, of Pittsford, N. Y., were present as visitors.

Minutes of last meeting were read and approved.

Sections 164 to 169 of the *Organon* were read, with discussion as follows:

Dr. Grant—The sections read are some of the most important and interesting of the *Organon*.

Dr. Schmitt—I think the sections read explain why there are cases where the high potencies do not act, and the low will. I do not agree with Dr. P. P. Wells, that a high potency will act if a low will. I make the point that we can have a remedy, according to Hahnemann, very similar to the case—not simillimum—where a single dose or repeated, of a high potency, will not do anything for you, but in a lower potency we get an effect, although transient, and not a cure. The case I will give will illustrate the point. It is a case of chronic diarrhœa where the indications were for Sulph. I gave it in the MM, CM, 200, in single and repeated doses, without any effect whatever, then gave the 30th potency, a dose for two or three mornings, which controlled the diarrhœa, but did not cure. I knew I had only the similar, not the simillimum; after a time the patient developed a cough, that came on at four A. M. with retching, blueness of the face, cold sweat, and *trembling*. Ant-tart.$^{cm}$, one dose cured cough and diarrhœa. Sulph. was the similar remedy and Ant-tart the simillimum.

Dr. Biegler—This case of Dr. Schmitt's does not invalidate the principle, that we must find the simillimum to cure. I think that his case helps to prove the fact, that *we must prescribe the simillimum to cure;* his stating that Dr. Wells never knew of a case that could not be relieved by a high potency, if the low would relieve, leads us to think there are exceptions; there are cases where the symptoms are obscured and do not come up until developed by some remedy, and I believe that Sulph. developed this case. The fact still stands, that disease is only cured by the true remedial agent (the simillimum), and will cure in a high potency; a low is not required.

Dr. Johnson—Does it not illustrate the palliative effect of remedies?

Dr. Biegler—I do not believe that a high potency will fail if it is the simillimum. I prescribed last year Rhus-tox, for a case of eczema, the indications I do not remember; it disappeared quickly, and I thought it was cured; a few weeks ago it came back worse than before, and in her letter, begging for the remedy of last year, without any indication of the present condition, I informed her it was a mistake to give the remedy of last year, but did send it, and asked her to write me a true description of her case, which she did, and I sent Pulsat. Among the chief indications were: want of fresh air; aggravation from cold drinks; this relieved for ten days, and to-day she sends me a letter, every indication calling for Sulph.

Another case, of a child seven weeks old, diarrhœa since birth; two weeks ago the stools began to be watery, gushing, and yellow; would saturate everything. I gave Podo. which relieved for a few days; then the mother's nipple became sore, with a shooting pain from nipple through to back. I gave Crot-tig. to the mother, with improvement in the child; in twenty-four hours there was a great disturbance, stools worse and more frequent, almost hourly. Sac-lac. Improvement followed, lasting three or four days, then worse, with the same yellow, watery, gushing stools, *with wind;* mother's nipple growing better.

I have known the mother a long time, also her father, and that there was sycosis in the family, the mother bears traces of it; three days ago I gave Thuja$^{cm}$, one dose, and to-day the child is much better; two stools last night, and up to noon to-day only one; if you will compare the stools of Thuja and Crot-tig. you will find it difficult to distinguish the difference between them. Here we have a case where it is difficult to select between two remedies that are similar to the case.

Dr. Grant—You gave the Crot-tig. to the mother and Thuja to the child?

Dr. Biegler—Yes; was tempted to give the Thuja to the mother, too, but for some reason I do not now remember I did not; this child was feeble from birth and remained so for over a month. We are assembled to learn of the best treatment for disease surrounded by those that shut their eyes, guided by the "scientific," and will not study the principles of Homœopathy; under their treatment the stools would have been checked, and the child would have died.

Dr. Grant—There is still another point, that if their treatment did not kill, if your supposition is correct, the suppression would complicate any sickness that would follow.

Dr. Biegler—This case illustrates how a remedy will come up, seem to be indicated, and not cure. It is not Homœopathy that is at fault.

Dr. Johnson—Can you get an aggravation from a high potency not the simillimum? The question was suggested by your statement of the action of Crot-tig.

Dr. Biegler—That is a great question; it looks to me that the disturbance that followed was caused by the remedy, although it was not the simillimum. In acute diseases not complicated with a miasm, we expect a cure after an aggravation.

Dr. Schmitt—Hahnemann says, that if you give a similar remedy you may get an aggravation of some symptoms, but not the true aggravation as from the simillimum.

Dr. Johnson—We are taught that when we get an aggravation we have the right remedy.

Dr. Biegler—I should add the other symptoms for which I prescribed Thuja: The nurse told me *that when the child sneezed a mass of mucus would be blown from the nose; child had a loose cough with much loose phlegm in the throat.* Under Thuja you have, "Blows out thick, gum mucus, mixed with blood and pus." There was no blood or pus in this case. "Much mucus in the throat, hawked up with difficulty."

Dr. Brownell—I have noticed the comparison is very close between the stools of Thuja and Crot-tig.

Dr. Schmitt—I do not think Crot-tig. has any wind with the stools.

Dr. Brownell—I would like to relate a case, where sugar in the urine is a persistent symptom; *foul taste in the mouth; cold sweat on the lumbar and sacral region, worse during stool;* burning of the soles of the feet; burning of the skin from the knees down, wants them rubbed, which does not relieve. Gravity of the urine was 1040 July 6th. Kali-bich. has the symptom, "sweat on the back during the effort of stool," also "large quantities of colorless urine." Under this remedy the sp. gr. was reduced to 1020, with no relief of other symptoms, except sweat was partially relieved. I gave Sulph. which relieved, but the gravity went up. There is no doubt in my mind that I did not have the simillimum. There is another remedy that has sweat on the lower region of the back, and he is now on that remedy—Plantago$^{1m}$.

Dr. Biegler—I would give weight to those remedies that have sweat in the region mentioned, and remedies for cold sweat—you will find them in Bœnninghausen or Allen on fevers.

Dr. Adams—In speaking of Thuja, it reminds me that "sweat on uncovered parts" is characteristic, and always leads me to that remedy.

Dr. Brownell—There is another symptom : he cannot sign his name if any one is near him, he is so nervous. Plantago seems to cover all of his symptoms better than any remedy I know of.

Dr. Biegler—There is another point from which we must look for help, and that is family dyscrasias. I was in consultation in a case of vomiting of pregnancy ; remedies relieved for a time, and Pulsat. better than any other ; several remedies were well chosen by the physician in charge, but none would hold the case.

I had been prescribing the past year for the sister of this lady, and had hard work to keep her from going down—had spent hours over the case. During her menses she would have a severe jerking pain over the right eye, would jerk the whole body ; she kept going down, looking pale and yellowish-green, difficult to nourish. I had inquired particularly about the menstrual flow, but without any light. After a few times, the mother told me that she had noticed that the color of the menses was green. On the head symptoms and green menses I gave Lac-can., with a very happy result ; there is nothing much to do now in the case. Now, when we attend the sister for vomiting of pregnancy, remedies do not relieve for any length of time. On looking over the case I found one symptom that pointed very strongly to Lac-can., and I mentioned this to the attending physician, together with the fact that this remedy had done so much for the sister. On questioning the mother, we found that this lady had been troubled with green menses too. Dr. Schmitt will tell you the result.

Dr. Schmitt—She was doing fairly well on the remedies given, and that night after we were there the husband came to my office, stating his wife was suffering with headache. *He had to hold her head so she might get relief ;* shooting pain up the spine and in the ovarian region. I sent Lac-can.$^{cm}$ one dose, which relieved *all* her symptoms, and during the last parade she wanted to ride down-street.

Dr. Biegler—This case illustrates how difficult it is sometimes to find the simillimum. Family dyscrasias need remedies, and we will often stumble until we know them.

I would like to read a letter I received from a physician of Albany, not a true homœopath, and my reply. Although it does not state the case in full, it gives it very well. An old gentleman of this city traveled to Albany, and there suffered with retention of urine from an enlarged prostate gland. The doctors there failed to relieve him, he called in a prominent member of the *old school*, who aspirated five times, then sent him home, no doubt thinking that he was going to die, with his physician of that city, who flung at me all of the "scientific nonsense" that had been used, such as Morphine, Cocaine, etc. I thought the best thing that could be done for the patient was first to rid him of his physician, so I assured him that he could take the first train home, as we would take care of the case.

"ALBANY, N. Y., Oct. 1st, 1888.

"MY DEAR DR. BIEGLER:—Mrs. G. H——, who just left my office, tells me that she is much improved since seeing you, and also tells me that the gentleman, whom our Albany doctors failed to relieve, has found relief at your skillful hands. To be more exact, she said 'you were very busy the day she saw you, and had a desperate case from Albany.' I knew of the case here and of the failure to relieve him, and inquired how he was, and she said 'that he was better and going to get well.'

Now I am an honest inquirer about high potencies and *not a scoffer*, and yet not a full believer. Would it be too much to ask you to drop me a line saying what remedy and what potency relieved such a desperate case?

"Very truly yours,

"GEO. E. G——."

"ROCHESTER, N. Y., Oct. 7th, 1888.

"GEO. E. G——, M. D.:—

"DEAR DOCTOR:—As it is often the case with me, several days elapse before I can find time to reply to letters, and I am disappointed in being in that situation since the receipt of your letter of the 1st inst. I gladly give the information you request concerning the case of Mr. Sperry, who was taken sick in Albany and returned to his home in care of Dr. J——.

"When I saw him on the night of his return, I found the urethra in such a condition as to prevent the insertion of the catheter. I then prescribed Nux vom.$^{200}$, for the purpose of doing away with the effects of the previous drugging—Morphine, Cocaine, etc., and toward morning, Dr. Carr, who had charge of the case, relieved him with the aspirator. The next morning I advised the use of the aspirator for another period of twenty-four hours, and prescribed Benzoic-acid$^{cm}$ one dose, the indications for this remedy, or rather for those upon which I selected it, you will find in Hering's *Guiding Symptoms*, also in the *Condensed Materia Medica*. In thirty-six hours after the dose of this remedy, I found the prostate gland retracted, I may say at least one-third from its former size; before that it seemed to fill the cavity of the pelvis; now at this time the flatus, which was before incarcerated, passed readily, and I also passed a No. 7 elastic catheter without difficulty; this catheter was retained in the bladder three days. After its removal, the urethra was very sensitive, and the following symptoms were very pronounced; unsatisfied feeling after micturition (by means of catheter); *urging continued for considerable time; the slightest touch with the fingers at the tip of the penis would give severe pain and bring on spasm.* Pain at tip of the penis, you will find under Cantharis, although not given as here described. We gave him Cantharis$^{cm}$ one dose dry on the tongue, and in twenty-four hours the symptoms for which it was given were entirely relieved. The patient now kept very comfortable, and gained in strength daily for four or five days, requiring the use of the catheter twice in twenty-four hours, during which time remedies were not given. At this time, however, fearing that the continued use of the catheter might be required, and not desiring to dance an indefinite attendance, I tried several elastic catheters preparatory to instructing him in their use, and by so doing I again inflamed, or at least congested the urethra to the extent of producing the following symptoms: *Urethra inflamed and sore along the whole length;* burning while urinating, *worse after; burning, smarting in the urethra in its whole length; jerking, stitching pain in the urethra.* For these symptoms we gave him Cannabis-sat.$^{cm}$ one dose dry on the tongue, and in solution, every four or six hours; all this was relieved, and he commenced to pass water naturally, so that the catheter was used only once in twenty-four hours for a few days, not necessarily, but as a matter of precaution to avoid accumulation in the bladder.

"In justice to myself I must say that I did not volunteer the information relating to the case to Mrs. H——, but that some one in my office spoke of it first; in fact, I am not aware of having said anything.

"I am thankful for your letter, as it is gratifying to come in contact with a man who is willing to inquire into the true method of cure, and when I do, I not only appreciate the honest disposition, but would do a great deal to help him, as I can well remember how I groped in the dark during the first ten years of my professional life, without a ray of light afforded from any source except my books.

"Let me now, for the present, ask you to cease looking through your microscope for the evidence of the remedial power of drugs and for the cause of disease, for there is nothing material about either—they are both imponderable powers; you doubt this I know, but do not say you will not believe before you have done what is only reasonable—that is, to investigate honestly and prove it to your satisfaction. In doing this you must conform to the law of cure, as it is exacting, and will tolerate no deviations; and I would here remind you that this law does not require a high dynamization, but absolutely the simillimum of selection to the case, and this cannot be done by multiple prescription.

"The alternation of remedies may sometimes be practiced, but that requires the ability of a master, and in ordinary practice it is destructive to an intelligent understanding of the peculiar or special action of remedies. As to the efficacy of the higher powers, this knowledge is obtained only when a thorough realization of the law is acquired; they are preferred by those only who, by long practice, have conformed to the law of cure. Since writing, I have called on Dr. Carr to ascertain the condition of the patient to date, and the result I can give you in a few words: *he is well*, passes his urine naturally and perfectly freely, which he had not done in the year past. He has had no other medicine since I discontinued my visits, which is now more than a week.

"Very truly yours,

"J. A. Biegler."

Dr. Brownell—Was there any history of gonorrhœa in the case?

Dr. Carr—None that I am aware of. I have known him a long time, and believe would have found out if there was any such condition. He now passes his urine better than for some years back, and the water is clearer. Seven or eight years ago he rode all day in the wet, and had an attack of inflammation of the prostate gland, from which he never fully recovered.

Dr. Schmitt—This is an immense case and should be published.

Dr. Biegler—The lady spoken of in the letter came to me for treatment from the hands of a mongrel, and I made the following notes from what she told me: A year ago she had sea-bathing which was followed by rheumatism, and has not been well since. She was dosed by a mongrel in Albany, who finally thought her anus needed attention, and this he stretched to the extent of nearly killing her. Then he thought that the uterus needed special attention, and in order to treat her "scientifically" he explored the uterus with a steel sound, and in this way he cut her so badly that he became alarmed, and finally peritonitis followed. After this she was treated by the additional assistance of another physician of "scientific notions," with ponderable doses of Quinine "for chills." Since then she has been dosed by all kinds of drugs.

Adjourned to Dr. Biegler's office in one month.

W. H. Baker, *Secretary.*

